# Personals

**Published:** 1916-07

**Authors:** 


### Personals.

Dr. Crandall, of Brownwood, has gone to New York to spend the summer in special study.

Dr. and Mrs. H. W. Harper, of Austin, have gone to Louise, Virginia, to spend the summer.

Dr. W. R. Sanderlin, of Graham, and Miss Carrie Newton, of Grandview, were married recently.

Dr. Homer Donald, of Dallas, has gone to Boston, where he will attend the Harvard Post-graduate School.

Dr. Howard Fitzgerald, of Eagle Lake, has gone to Chicago to take a six or eight weeks' post-graduate course.

Dr. and Mrs. Frederick Duncalf, of Austin, will go to Battle Creek, Michigan, to spend their summer vacation.

Dr. C. J. McCormick, of Fort Worth, has returned from Kansas City, where he has been specializing in dentistry.

Dr. C. E. Johnson, of Seymour, has returned from Chicago, where he has been taking a past master's course in baby diseases.

Dr. S. J. Pate, of Beaumont, who has been away for several weeks taking a post-graduate course in New York City, has returned home.

Dr. M. W. Pitts, of Luling, who has been taking special work for the past few weeks at the Tulane University in New Orleans, returned home Friday afternoon.

Dr. E. C. Beaumont, of San Saba, has returned from New Orleans, where he has spent several weeks taking a course of lectures.

